# Chasing a Little-Known Fairy Bee (*Perdita meconis*) in a Dynamic Desert Landscape

**DOI:** 10.3390/insects15110892

**Published:** 2024-11-14

**Authors:** Sarit Chanprame, Colleen Meidt, Terry Griswold, Joseph S. Wilson, Kelsey K. Graham

**Affiliations:** 1 Department of Biology, Utah State University, Logan, UT 84322, USAcolleen.meidt@usu.edu (C.M.); 2 USDA-ARS Pollinating Insects Research Unit, Logan, UT 84322, USA; terry.griswold@usda.gov (T.G.); kelsey.graham@usda.gov (K.K.G.); 3 Department of Biology, Utah State University-Tooele, Tooele, UT 84074, USA

**Keywords:** Las Vegas bearpoppy, *Arctomecon*, pollination, conservation, bee decline, Mojave poppy bee

## Abstract

The Mojave poppy bee, *Perdita meconis* Griswold, is a small fairy bee endemic to the eastern Mojave Desert, known to be associated with the Las Vegas Bearpoppy, *Arctomecon californica*. Because of the reported decline in populations of *P. meconis,* it has been targeted for protection under the Endangered Species Act. Here, we present the results of a 3-year study investigating the current distribution and floral preferences of *P. meconis* in Clark County, Nevada. We found that populations of *P. meconis* fluctuated dramatically from 2020 to 2022. This area experienced extreme drought conditions during these years, which consequentially affected the populations of this rare bee and its floral resources. Analyses of the pollen collected from *P. meconis* demonstrated fidelity to *Arctomecon californica*; over 90% of the pollen extracted from the body of these bees and nearly 100% of the pollen carried on female scopae was from *Arctomecon californica*, confirming this bee as a pollen specialist. Our data also show that *P. meconis* likely visits *Psorothamnus*, perhaps as a nectar source, since *A. californica* does not produce nectar. These data can aid conservation efforts for the Mojave poppy bee, providing valuable insights on the distribution and floral requirements of this bee that can assist conservation efforts.

## Simple Summary

Growing concerns about the decline of bees worldwide have led scientists to increase their efforts to better understand where individual bee species live and what their biological needs are. Here, we investigate the current distribution and behavior of a rare bee, the Mojave poppy bee. This bee is being assessed for protection under the Endangered Species Act, yet little is known about its current distribution and what its floral needs are. Our 3-year study found that populations of the Mojave poppy bee fluctuated widely across three consecutive years, 2020–2022. Furthermore, we detected it in fewer locations than were previously known. To learn more about the flowers it visits, we conducted pollen analysis on the pollen found on the bodies of these bees. We found that most of the pollen was from the Las Vegas Bearpoppy, confirming that this bee is a poppy specialist. We also found some pollen from indigo bush blooms on the bees, suggesting this shrub might be an important nectar source for these rare bees.

## 1. Introduction

It is widely understood that bees play an important role in many different ecosystems, due to the services they provide as pollinators [1,2]. Wild native bees are critical not only for wildlands, but also for many agricultural systems [3]. There is a growing consensus that some bee communities and bee species are experiencing a decline [4,5,6,7], although data are not available for most native species. This limitation inhibits our ability to make informed conservation decisions.

The Mojave poppy bee, *Perdita meconis* Griswold (Andrenidae: Panurginae), a species of fairy bee restricted to the eastern Mojave Desert [8], is one such poorly known bee. Concern has been raised about the viability of this species, as its populations appear to have dramatically declined in recent years [9]. Due to the reported declines, a petition was made to federally list *P. meconis* as an endangered species in 2018 [10]. Crucial to the decision-making process is a better understanding of the current distribution of this bee and its life history traits, especially those related to floral resources.

*Perdita meconis* is colloquially known as the Mojave poppy bee, because it is endemic to the eastern Mojave Desert, and its specific name references a floral association with poppies of the genus *Arctomecon* Torrey and Fremont (Papaveraceae). Like most fairy bees, *P. meconis* is small, only 5–7 mm long. Based on its size, a predictive model suggests that these bees are not strong fliers, with foraging ranges estimated to be just 100–500 m [11]. Adult *P. meconis* are active for a short period of time in the spring, approximately from the middle of April to the beginning of June [12]. This coincides with much of the blooming season of its host plants.

The principal host plants of *P. meconis* are bear poppies (*A. californica, A. humilis*, and potentially *A. merriamii*) and the prickly poppy, *Argemone.* It is important to note that the known host plants, *Arctomecon* and *Argemone,* do not produce nectar [13], and it remains unknown where *P. meconis* might forage for nectar. In Clark County, Nevada, where the majority of *P. meconis* populations are known to be found [9], *A. californica* (the Las Vegas Bearpoppy) is the principal host plant providing pollen to these bees. It is a rare herbaceous plant, endemic to the eastern Mojave Desert, most frequently found in association with highly alkaline gypsiferous soil [14]; one of the few plants able to tolerate such an environment. Thus, populations of *A. californica* act as islands of resources, dotting the desert landscape, largely devoid of other plants, and *A. californica* are known to attract diverse floral visitors [12]. Its distribution and reproduction were initially studied in the 1990s [15], a time of abundance for the plant that peaked in 1993 [16]. Populations have been declining ever since [12,17]. The plant’s short lifespan [12,18], combined with sporadic germination [19] and seedling establishment, results in transient habitat islands, increasing the extinction risk for *P. meconis* given its suspected weak flight capability.

The interconnected relationship between these two rare and potentially at risk taxa, *A. californica* and *P. meconis*, was the focus of this study. Our objectives were to: (1) determine the current distribution of *P. meconis* in Clark County, Nevada; (2) test its dependence on the Las Vegas Bearpoppy by performing pollen analyses on *P. meconis* samples; and (3) identify potential plants that might provide nectar resources for *P. meconis*.

## 2. Materials and Methods

### 2.1. Study Sites

**Figure 1:**
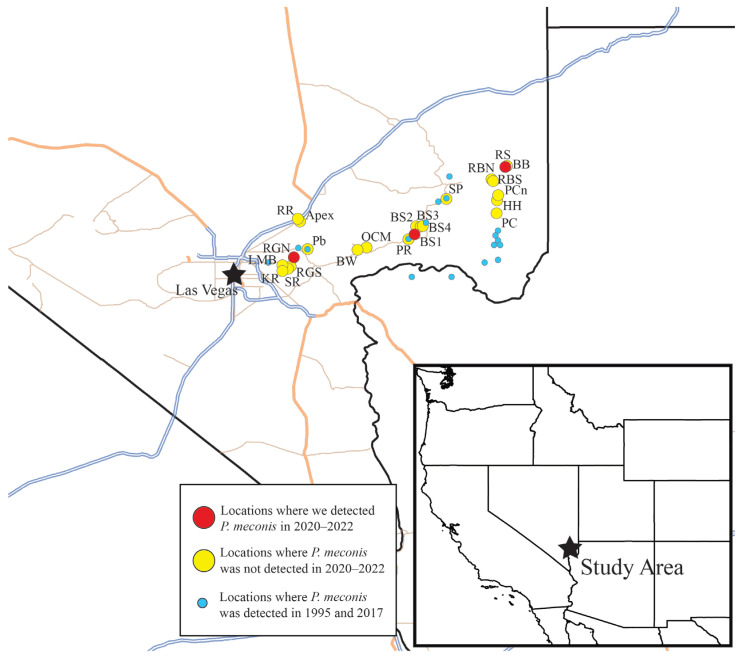
Map of *P. meconis* distribution, historically (small dots) and detected in 2020–2022 surveys (large red dots). Sites where the host plant was present, but *P. meconis* was not (large yellow dots).

The study sites in Clark County, Nevada, were selected based on documented historic sites of *A. californica* and *Argemone* [9,15], new populations reported by BLM and USFWS contacts, and sites discovered by field crews while traveling to selected sites during the surveying period. Twenty-three sites were visited during 2020–2022 (Table 1, Figure 1).

#### 2.1.1. Site Selection 2020

While many *A. californica* populations were in bloom in 2020, only seven sites were sampled due to the travel restrictions imposed due to the COVID-19 pandemic, which were all located on land managed by the Bureau of Land Management (BLM).

#### 2.1.2. Site Selection 2021

Sixteen sites were surveyed in 2021, fourteen sites with *A. californica* populations and two with *Argemone.* The fourteen *A. californica* sites were chosen based on surveys from the previous year and historical records on *A. californica* populations [9,15]. These sites were then surveyed during the first week of the field season to determine their potential for standardized surveys, based on the number of potentially flowering plants, the density of the plant population, and the documentation on historical *P. meconis* presence.

The selected sites were categorized into two groups: pollinator survey sites and low-priority/opportunistic collection sites. The three low-priority sites, Ore Car Mine (OCM), Borax Wash (BW), and Poppy Canyon (PCn), were visited infrequently for opportunistic collection of *A. californica*. OCM had only two living plants and BW had fewer than five potentially blooming plants. While PCn had moderate numbers of *A. californica*, it was relegated to low priority because the steep terrain meant there was no way to safely and thoroughly survey the site without greatly disturbing the exceptionally well-developed cryptobiotic crust. Ten sites were selected as pollinator survey sites (Table 1). All but RS had a moderate to high density of *A. californica*. RS was included due to the detection of *P. meconis* in 2020. These sites were visited on a biweekly basis to carry out pollinator surveys (see below for survey protocols).

In addition to *A. californica*, two sites of *Argemone* were discovered during the initial site visits. Lake Mead Boulevard (LMB) consisted of six blooming plants, while Kodachrome Road (KR) had a single blooming plant on the edge of the gravel road. Both sites were surveyed opportunistically for *P. meconis.*

#### 2.1.3. Site Selection 2022

Fifteen *A. californica* sites were selected after the first week of preliminary surveys, to determine the suitability of the sites visited during the previous years. As in 2021, the sites were categorized into two groups, pollinator surveys sites and low-priority/opportunistic collection sites. Of the twelve pollinator survey sites, eight were the same as 2021 (Apex, BS2, Pb, PC, RBN, RBS, RR, RS). RS was again included within this category due to presence of *P. meconis* samples in 2020. The new pollinator survey sites were Black Butte (BB), Bitter Spring 1, Helicopter Hill, and Stewart Point. BB was newly discovered and was included due to the proximity of the site to RS, although neither of these two sites had strong populations of *A. californica*. Three new low-priority sites were added: Bitter Spring 3 (BS3), Bitter Spring 4 (BS4), and Pinto Ridge (PR). Because *P. meconis* had been reported as being present at PR in the past [9], it was included as a low priority site due to few plants being present with limited flowering, along with hazardous terrain. One of the *Argemone* sites, LMB, was again surveyed opportunistically due to the presence of five blooming plants; KR was removed because of the absence of plants.

### 2.2. Survey Methods

Surveys for *P. meconis* were conducted from 2020 to 2023, during the blooming season of its floral hosts (roughly March through May). Surveys were conducted, from slightly before the expected flight time of *P. meconis* to near the end of the known flight period. While surveys focused on flowering *A. californica* and *Argemone*, pollinators on other co-flowering plants were also collected to confirm the assumed specialization of *P. meconis*.

A combination of timed visual observations and net collections were used to encompass the temporal and spatial coverage of the host plants during the blooming season. Visual observations were used to identify the presence of *Pygoperdita* at the survey sites. Field crews were trained to distinguish *Perdita* (*Pygoperdita*), the subgenus that includes *P. meconis,* and another related poppy specialist, *P. robustula*, from other floral visitors, using a combination of profile images and museum specimens. *Perdita meconis* and *P. robustula* are similar in size and appearance, making field identification unreliable. The sites where we conducted visual observations were then prioritized for repeated surveys across the spring season. Net collections were used to confirm the presence of *P. meconis,* by collecting vouchers. As *P. meconis* is considered rare, precautions were taken to ensure a minimal impact of the study on the population. Since a significant proportion of males do not contribute to reproduction in bees [20], specimen collections were restricted to males to minimize the impact on local populations. The only exception was when we found *Perdita* that were prey of crab spiders (Thomisidae); these females were collected and recorded separately. *Perdita* specimens were curated and identified in the US National Pollinating Insects Collection.

#### 2.2.1. Visual Observation

Visual observations began as soon as the first poppy plants bloomed, which depended on the elevation and exposure. These observations consisted of an observer visiting five *A. californica* plants spread across a survey site and conducting 5 min observations of each plant. During each visit there were two observers, thus totaling 10 plants and 50 min per observation event. Environmental data, such as the site name, date, time, weather conditions, wind speed, and temperature, were recorded at the initiation of each observation period. At the start of each observation of an individual plant, the observer positioned themselves approximately one meter away from the plant, putting the flowering plant between themselves and the sun, so as not to cast a shadow on the plant. Plant specific data, such as the number of flowers, buds, and fruits present, were recorded. The observer would then record the pollinators visiting each plant at the genus level and when possible, the species, with minimal interference in terms of the pollinator’s activity.

#### 2.2.2. Net Collection

At the initiation of net collections, the date, time, temperature, and wind speed were recorded. Each collector visited 50 flowering *A. californica* plants for each poppy population or, if a population was smaller, recorded the total number of flowering plants visited. Each collector would spend 30 s at each of the 50 flowering plants and collect all the pollinators present. Net collections were made carefully to avoid damage to the plants. After 30 s at a plant, the collector would move to the next flowering plant and collect for another 30 s. With two collectors each vising 50 plants for 30 s each, a total of 50 person/minutes were spent collecting at each site. While collecting, the collectors also inspected the blossoms for any crab spiders with *Pygoperdita* prey and collected those bee specimens as well.

### 2.3. Pollen Analyses

#### 2.3.1. Pollen Library Preparation

A pollen library of plants co-flowering with *A. californica* populations was created. In total, 16 species of flowering plants found in proximity to *A. californica* populations in Clark County were sampled for pollen. The anthers were collected in centrifuge tubes and suspended in 70% ethanol for preservation. The centrifuge tubes were sonicated to release pollen grains from the anthers. Each pollen solution was pipetted out and deposited onto a slide and placed on a heating plate in order for evaporation to take place. Fuchsin dye gel [21] was added and melted onto the slide to accentuate the pollen features. The pollen slides were studied under 400× (and 1000×) magnification. The appearance of the pollen was noted for further comparison with the pollen slides from *P. meconis* specimens.

#### 2.3.2. Pollen Sampling

Pollen was collected from representative *P. meconis* males and females from 2020–2022 collections, by dabbing a small piece of Fuchsin gel on the tip of an insect pin onto the dorsum of the thorax, an area where pollen tends to collect because it is difficult for bees to groom themselves, resulting in pollen grain build-up [15]. Pollen slides were made from these samples, by allowing the fuchsin gel to melt onto a slide using a hot plate.

For female *P. meconis* with pollen loads, the pollen was removed from one scopa. The pollen was suspended in 70% ethanol and sonicated to loosen the pollen grains. Pollen slides were then made, the same way as for the pollen library.

In regard to pollen counts, mounted pollen slides are first viewed under 100× magnification on a digital scope. The slide is divided into a 4 × 4 sequentially numbered grid. A random number generator is used to select the grid cells. Pollen counts by kind (identified by comparison with the pollen library), per cell, are recorded until the total cell count exceeds 500.

#### 2.3.3. Surveys on Co-Flowering Plants

To further explore floral specialization in *P. meconis* in 2020 and 2021 at the sites where there were co-flowering plants, bees were also collected. At some survey sites bear poppies were the only plants in flower, because the gypsiferous soil that bear poppies inhabit is tolerated by only a few flowering plants.

#### 2.3.4. Museum Floral Records

The specimen data for *P. meconis* specimens were mined from the collection of the USDA-ARS Pollinating Insect Research Unit (BBSL) for floral associations, to further investigate the floral affinity of *P. meconis* to poppies.

## 3. Results

### 3.1. Perdita Meconis Surveys

#### 3.1.1. 2020 Surveys

Eight sites (Table 2) were surveyed between 29 April and 14 May, with a total of 40 collection events. Perdita meconis was detected at three sites, namely BS1, RGN, and RS, with the highest abundance at BS1. The surveys detected 31 specimens at BS1, 23 specimens at RS, and 6 at RGN (Figure 1). All of the *P. meconis* individuals collected in 2020 were visiting *A. californica*. Although they were not present at all the sites surveyed, where they were present, *P. meconis* individuals seemed to be the most abundant *A. californica* visitors.

#### 3.1.2. 2021 Surveys

Sixteen sites were surveyed between 8 March and 7 May. A total of 66 collection events occurred across all the sites. Visitor activity was low throughout the survey period. No *P. meconis* were detected at any site, despite a total of 55 collector hours spent searching for *P. meconis* (50 min visual observations at each collection event).

#### 3.1.3. 2022 Surveys

In 2022, 16 sites were surveyed from 30 March to 13 May. A total of 61 collection events occurred across all the sites. Only two *P. meconis* individuals were collected, one male from BS1 and one female from RS (Figure 1), with a similar level of effort to 2021, over 50 collector hours spent searching for *P. meconis*.

### 3.2. Pollen Analysis

**Figure 2:**
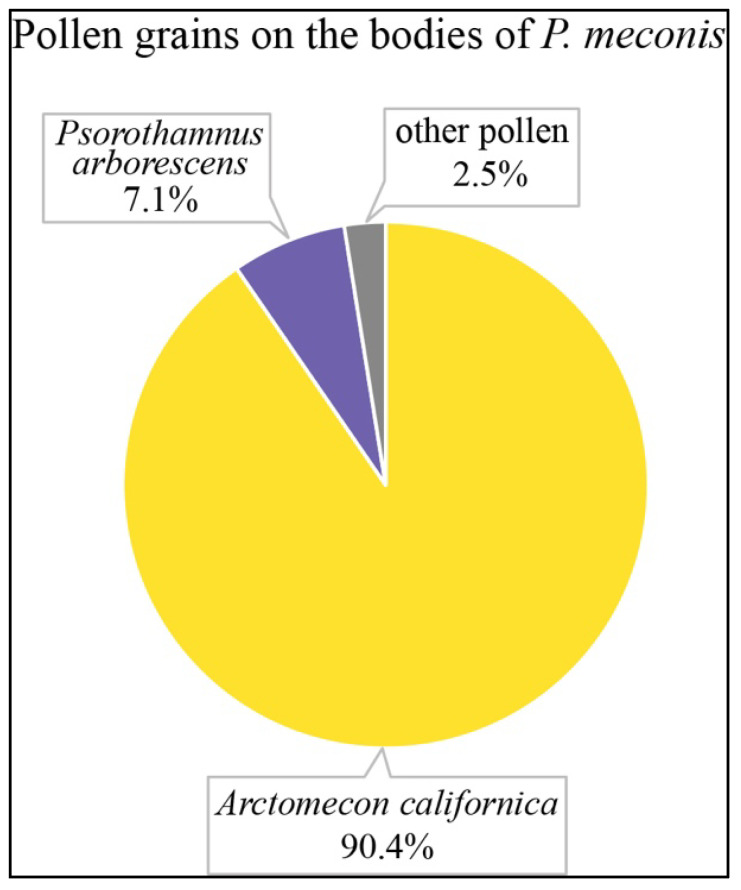
Results of our pollen analysis of pollen grains removed from the bodies of *P. meconis* specimens (*n* = 23).

Pollen analyses were performed on 23 specimens of *P. meconis* collected in 2020 (21 males, 2 females) and the two specimens collected in 2022 (1 male, 1 female). Pollen taken from the thoracic dorsum of *P. meconis* consisted largely of *A. californica* pollen (90.4%). Fourteen additional pollen types were identified, of which *Psorothamnus arborescens* (Fabaceae, (A. Gray) Barneby) was the most common (7.1%) (Figure 2). Other pollen detected included *Phacelia parishii* (Hydrophyllaceae, A. Gray), *Stanleya pinnata* (Brassicaceae, (Pursh) Britton)*, Eschscholtzia glyptosperma* (Papaveraceae, Greene), *Brassica tournefortii* (Brassicaceae, Gouan), *Cylindropuntia ramosissima* (Cactaceae, (Engelm.) F. M. Knuth)*, Chylismia brevipes* (Onagraceae, (A. Gray) Small), *Baileya multiradiata* (Asteraceae, Harv. and A. Gray ex Torr.), *Eriophyllum lanosum* (Asteraceae, (A. Gray) A. Gray), *Encelia farinosa* (Asteraceae, A. Gray ex Torr.), *Xylorhiza tortifolia* (Asteraceae, (Torr. and A. Gray) Greene), *Sphaeralcea ambigua* (Malvaceae, A. Gray), and two unknown plants in minute amounts (<1% of pollen grains).

Pollen taken from the scopa of *P. meconis* females was in the form of a packed ball and retained its shape after removal from the bee’s leg. The pollen grains stayed attached to each other and became large fragments when the pollen ball was broken down. These observations support the classification of *P. meconis* as wet packing their provisions rather than dry packing [22]. Essentially all of the pollen from the scopal loads of *P. meconis* was from *A. californica* (99.6%). Trace amounts of other pollen were from *Psorothamnus arborescens* (0.16%), *Phacelia parishii* (0.16%), and *Cylindropuntia ramosissima* (0.08%).

### 3.3. Museum Floral Data

The BBSL collection houses 243 *P. meconis* specimens of which most were collected on the poppy genera, *Arctomecon* (N = 127) and *Argemone* (N = 88). Singletons (N = 6) were associated with *Calochortus* sp. (Liliaceae), *Psorothamnus arborescens* (A. Gray) Barneby (Fabaceae), *Sphaeralcea ambigua* A. Gray (Malvaceae), *Enceliopsis argophylla* (D.C. Eaton) A. Nelson (Asteraceae), and *Chylismia brevipes* (A. Gray) Small (Onagraceae). The remaining 22 specimens in the museum were collected in pan traps and have no floral records.

## 4. Discussion

### 4.1. Distribution of P. meconis in Clark County

Across three years of surveys (2020–2022), *P. meconis* was only detected at three sites: BS1, RGN, and RS (Figure 1). The number of *P. meconis* individuals varied greatly from year to year, with approximately 60 specimens collected in 2020, none the following year, and only two specimens collected in 2022. This dramatic decline in *P. meconis* could be influenced in part by the decline of their host plant, *A. californica*. Throughout the years assessed in this study, the eastern Mojave Desert experienced intense drought conditions, which led to large fluctuations from year to year in the number of plants that bloomed and survived between the years [12]. *Arctomecon californica* seeds require a very specific environment to germinate [19], which results in cohorts of seedlings germinating in appropriate conditions. These seedlings will have a similar growth rate and reach the end of their lifespans at a similar time, resulting in transient patches of *A. californica* that exist for approximately a decade and then disappear, leaving a seed bank in the soil waiting for good conditions to spring back up again [18,23,24].

The duration of the drought may have been detrimental to many of the historically vibrant poppy populations. Of the 11 sites where *P. meconis* was observed visiting poppies in 1995 [15], and again in 2017 [9], only two (Pb and SP) had living *A. californica* plants in the years assessed in our study. Furthermore, no *P. meconis* were detected at either of these two sites during our study. The more recent report from 2017 [9] indicated that Pb (PR in the cited publication) had a large population of *P. meconis* and SP (SB in the cited publication) had a moderate population of *P. meconis*. The report also indicated that there were only minor changes in the poppy and bee populations between the surveys conducted in 1995 and 2017. This contrasts greatly with our findings. While many populations of *A. californica* had disappeared, our study found that the poppy population at Pb showed little change in terms of plant numbers, compared to the 2017 survey. Despite the apparently stable flowering plant populations, no *P. meconis* individuals were detected.

Our survey data vary greatly from year to year in our 3-year study. In 2020, *P. meconis* individuals were found in healthy numbers at BS1, RGN, and RS. Because of the 2020 findings, these three sites were intensely surveyed in the following blooming seasons using the same protocols, yet *P. meconis* individuals were not detected in 2021 and, in 2022, only single individuals of *P. meconis* were detected at BS1 and RS. Given the robust populations in 2020, it is unlikely that our inability to find *P. meconis* in subsequent years was due to a failure in effort. Furthermore, *P. robustula*, which is very similar to *P. meconis*, was found at multiple sites in all three years assessed in our study. Rather than local extinction explaining our inability to detect the Mojave poppy bee, it is likely that *P. meconis* is able to suspend its development (diapause) and remain underground during unfavorable conditions, possibly for several years. Many native xeric bee species are suspected to be able to remain in diapause until favorable conditions return [25,26,27], with some bees able to stay in diapause for several years [28]. For example, a closely related bee, *Macrotera portalis* Timberlake, described originally as a *Perdita,* can remain in diapause for at least three years [20]. This diapause adaptation likely also occurs in *P. meconis*, whose survival depends upon the success and survival of its host plants. We suggest diapause in *P. meconis* populations could explain our inability to detect it during 2021 and only minimally in 2022, both years where drought conditions were extreme. We suggest that further surveys of areas with historical *P. meconis* records are needed to gain a more thorough understanding of the current status of this imperiled species.

### 4.2. Pollen Analysis of P. meconis

Though 15 types of pollen were found on the bodies of *P. meconis*, the results from the pollen analyses reinforce the oligolectic status of this bee, with approximately 90% of the pollen grains present on their bodies from *A. californica* (Figure 2) and essentially 100% of pollen in female scopal loads from *A. californica*. While *P. meconis* does seem to specialize on poppy pollen, the fact that over 7% of the pollen extracted from the bees’ bodies belonged to *P. arborescens* suggests this plant might also play a role in the biology of the Mojave poppy bee. Pollen from *P. arborescens* was found on 24 of the 25 bees we analyzed, suggesting that this legume may be a source of nectar for *P. meconis. Psorothamnus arborescens* was relatively common at many survey sites and was found in bloom across the sampling years. The third most abundant pollen type from the analysis was identified as likely coming from *Eriophyllum lanosum*, which also offers nectar and is relatively common in the area. 

It should be noted that there is a possibility that these rare pollens are from nectar visits or are simply contaminants from the collecting process. Contamination could come from three sources: foreign pollens previously deposited on *A. californica* flowers by generalist bees, pollen picked up while inside the sampling vials, or pollen picked up while inside the net. We suggest that of these three possible sources of contamination, pollen picked up from inside the sampling vial is the most likely source. The field collection protocol used in this survey required all specimens from each type of host plant in the same location to be stored in the same sampling vial. Many generalist bees, such as the *Apis mellifera* and *Lasioglossum* species were collected from *A. californica* [12] and were placed together with *P. meconis* inside the same vial. However, the fact that *P. arborescens* pollen was found on nearly all of the bees we examined leads us to believe that it does represent an important plant in the ecology of these bees, not just a contaminant from the collection process.

As we build an understanding of *P. meconis* life history traits, it is important to recognize that nesting behavior and nectar sources are still knowledge gaps. For the latter, our results suggest *P. arborescens* (Mojave indigo bush) as a candidate nectar source, which is in need of confirmation.

## Figures and Tables

**Table 1:** Locations of *Arctomecon californica* and *Argemone* that were surveyed during 2020–2022, in alphabetical order by code (see Figure 1).

Site Name	Code	Location	Radius (m)
Apex	Apex	36.305500, −114.939444	500
Black Butte	BB	36.499921, −114.201760	100
Bitter Spring 1	BS1	36.258327, −114.528040	300
Bitter Spring 2	BS2	36.287710, −114.522428	500
Bitter Spring 3	BS3	36.286681, −114.505937	500
Bitter Spring 4	BS4	36.288654, −114.499012	300
Borax Wash	BW	36.203463, −114.732925	300
Helicopter Hill	HH	36.381543, −114.231045	600
Kodachrome Rd	KR	36.10344, −114.9911060	50
Lake Mead Blv	LMB	36.199547, −115.000189	50
Ore Car Mine	OCM	36.212040, −114.700568	300
Pabco	Pb	36.205775, −114.912158	300
Poppy City	PC	36.335103, −114.233219	500
Poppy Canyon	PCn	36.398943, −114.227713	300
Pinto Ridge	PR	36.241706, −114.550132	200
Red Bluff Spring North	RBN	36.456679, −114.251431	300
Red Bluff Spring South	RBS	36.449705, −114.245201	500
Rainbow Gardens North	RGN	36.176468, −114.961672	400
Rainbow Gardens South	RGS	36.144443, −114.972828	700
Railroad	RR	36.314672, −114.947205	300
Restoration Site	RS	36.504502, −114.195595	300
Steward Point	SP	36.386026, −114.413172	200
Shooting Range	SR	36.135997, −114.981891	500

**Table 2:** Sites surveyed by year. Sites visited in 2020 are marked with X, indicating the site was visited. The following years sites were either visited following the PSs (pollinator surveys) protocol or the LP (low-priority sites) protocol (see methods for description of protocols).

Site Name	Code	2020	2021	2022
Apex	Apex		PSs	PSs
Black Butte	BB			PSs
Bitter Spring 1	BS1	X		PSs
Bitter Spring 2	BS2		PSs	PSs
Bitter Spring 3	BS3			LP
Bitter Spring 4	BS4			LP
Borax Wash	BW	X	LP	
Helicopter Hill	HH	X		PSs
Kodachrome Rd	KR		LP	
Lake Mead Blv	LMB		LP	LP
Ore Car Mine	OCM		LP	
Pabco	Pb		PSs	PSs
Poppy City	PC	X	PSs	PSs
Poppy Canyon	PCn	X	LP	
Pinto Ridge	PR			LP
Red Bluff Spring North	RBN		PSs	PSs
Red Bluff Spring South	RBS		PSs	PSs
Rainbow Gardens North	RGN	X	PSs	
Rainbow Gardens South	RGS		PSs	
Railroad	RR		PSs	PSs
Restoration Site	RS	X	PSs	PSs
Steward Point	SP			PSs
Shooting Range	SR		PSs	
Total Sites Sampled		7	16	16

## Data Availability

The data are presented in the manuscript. All specimens are housed in the U.S. National Pollinating Insects Collection, USDA-ARS Pollinating Insect Research Unit (PIRU), Logan, Utah.
